# Experiences of leadership in health care in sub-Saharan Africa

**DOI:** 10.1186/1478-4491-10-33

**Published:** 2012-09-13

**Authors:** Leslie Curry, Lauren Taylor, Peggy Guey-Chi Chen, Elizabeth Bradley

**Affiliations:** 1 Yale Global Health Leadership Institute, Yale School of Public Health, 60 College Street, PO Box 208034, New Haven, CT, USA; 2 RAND Corporation, 1776 Main Street, Santa Monica, CA, 90407, USA

## Abstract

**Background:**

Leadership is widely regarded as central to effective health-care systems, and resources are increasingly devoted to the cultivation of strong health-care leadership. Nevertheless, the literature regarding leadership capacity building has been developed primarily in the context of high-income settings. Less research has been done on leadership in low-income settings, including sub-Saharan Africa, particularly in health care, with attention to historical, political and sociocultural context. We sought to characterize the experiences of individuals in key health-care leadership roles in sub-Saharan Africa.

**Methods:**

We conducted a qualitative study using in-person interviews with individuals (n = 17) in health-care leadership roles in four countries in sub-Saharan Africa: the Federal Democratic Republic of Ethiopia, the Republic of Ghana, the Republic of Liberia and the Republic of Rwanda. Individuals were identified by their country’s minister of health as key leaders in the health sector and were nominated to serve as delegates to a global health leadership conference in June 2010, at Yale University in the United States. Interviews were audio recorded and professionally transcribed. Data analysis was performed by a five-person multidisciplinary team using the constant comparative method, facilitated by ATLAS.ti 5.0 software.

**Results:**

Five key themes emerged as important to participants in their leadership roles: having an aspirational, value-based vision for improving the future health of the country, being self-aware and having the ability to identify and use complementary skills of others, tending to relationships, using data in decision making, and sustaining a commitment to learning.

**Conclusions:**

Current models of leadership capacity building address the need for core technical and management competencies. While these competencies are important, skills relevant to managing relationships are also critical in the sub-Saharan African context. Developing such skills may require more time and a deeper level of engagement and collaboration than is typically invested in efforts to strengthen health systems.

## Background

Leadership is widely regarded as central to effective health-care systems [1], and is one of the World Health Organization (WHO) Health Systems Building Blocks [2]. Calls to strengthen national leadership in global health, such as in the Accra Agenda for Action [3], and the African Leadership Forum [4], are common. Resources are increasingly devoted to the cultivation of strong health-care leadership globally, including in low-income countries [5]. Core leadership competencies in health care have been identified and many models of competency-based education and training exist [6].

Nevertheless, the literature on theories of leadership [7] and models of leadership capacity building has been developed primarily in the context of high-income settings. Less research has been done on leadership in low-income settings [8-10], including sub-Saharan Africa. Despite the argument that consideration of historical, political and sociocultural context is essential to the conceptualization of African leadership [11-14], little empirical research has been conducted on leadership, particularly in the area of health.

Accordingly, we sought to characterize the experiences of individuals in key health-care leadership roles in sub-Saharan Africa. We used a qualitative approach to generate insights inductively. Understanding the experiences of leadership from the perspective of those in the leadership role can contribute to our conceptualization of leadership in healthcare in the African context. Insights from this study can be useful in informing, designing and implementing effective efforts to develop health-systems leadership capacity in the region.

## Methods

### Study design and setting

We conducted a qualitative study using in-depth, in-person interviews [15] with an information-rich, purposeful sample [16] of individuals in health-care leadership roles. A purposeful sample includes individuals who have direct experience with the phenomenon of interest and therefore can provide unique insight into the central research question. We contacted the Minister of Health in each of the four countries; each Minister identified individuals who serve in key leadership roles in the health sector at the national or regional level in each country. These individuals were nominated to serve as delegates to a global health leadership conference in June 2010, at Yale University in New Haven, Connecticut. We invited 18 individuals to participate; 2 declined, and 1 additional person volunteered, resulting in a final sample size of 17.

Participants were from four countries in sub-Saharan Africa: the Federal Democratic Republic of Ethiopia, the Republic of Ghana, the Republic of Liberia and the Republic of Rwanda Although these four countries represent considerable geographic diversity, they also share a number of key contextual factors. These factors include complex political conditions punctuated by occasional conflict, challenges of poverty such as famine and drought, under-resourced health-care systems characterized by a profound lack of human resources, inadequate supply chains and physical infrastructure, and poor financing systems. Finally, the selected countries also face the challenge of a general lack of public confidence in both the health-care systems and the government.

Despite these contextual challenges, each of these countries has demonstrated substantial recent improvements in their health delivery systems or health outcomes. For instance, at the time of this study (2010), Ethiopia had achieved a massive scale-up of health extension workers and remarkable reductions in malaria rates and infant mortality. Ghana had accomplished dramatic improvements in maternal mortality. Liberia had established a universal package of health services less than a decade after civil war. Rwanda had demonstrated the fastest documented decline in infant mortality rates in global health. The Human Investigation Committee at the Yale University School of Medicine in New Haven, CT, USA approved the research protocol.

### Data collection

**Figure 1:**
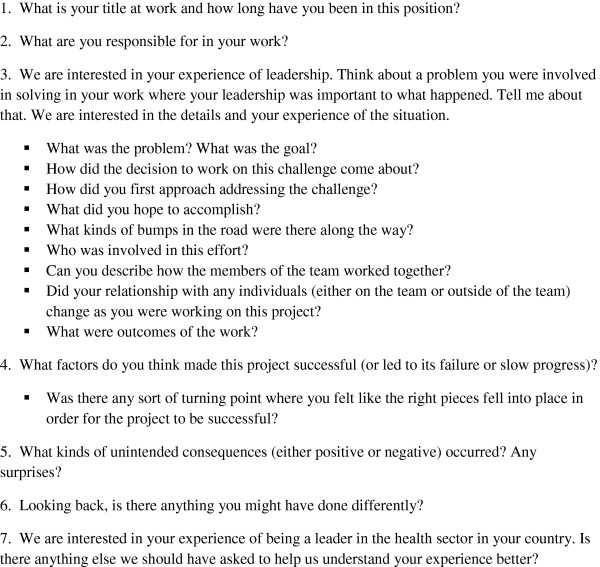
Discussion guide questions.

Interviews were conducted in English by researchers with training and experience in qualitative interviewing, and took place at the conference site. Interviews were typically 1 hour in duration and followed a discussion guide consisting of seven open-ended questions with probes to encourage clarification and elaboration as needed (Figure 1) [15-17]. Interviews were audio-taped, professionally transcribed and reviewed to ensure accuracy.

### Analysis

Data analysis was performed by a five-person multidisciplinary team with backgrounds in public health, medicine, humanities, and health services research. We conducted the analysis in stages and in accordance with principles of grounded theory [18], using systematic, inductive procedures to generate insights grounded in the views expressed by study participants. We employed the constant comparative method [17,18], an analytic approach in which verbatim quotes are catalogued into essential concepts (or codes). As the transcripts are reviewed, sections are constantly compared with previously coded sections to determine whether the same concept is apparent. If the concept cannot be classified within existing codes, the definition of codes may be expanded or refined as necessary to fit the concepts emerging from the data. Team members independently coded transcripts, meeting regularly to achieve consensus throughout the process. We finalized a comprehensive code structure (a list of codes and their essential properties and definitions) capturing all data concepts, which was then systematically re-applied to all transcripts [19,20]. We reviewed the final codes, including their defining properties and their relationship to each other, to arrive at consensus regarding the central, unifying themes that emerged from the data [16,19]. We used qualitative analysis software (ATLAS.ti 5.0, Scientific Software Development, Berlin, Germany) to facilitate data organization and retrieval.

## Results

Participants (n = 17) were diverse in gender, background, and the types of roles or positions they held within their country’s health-care system (Table 1).

**Table 1:** Participant characteristics

	**Number of participants (n = 17)**
**Country**	
Ethiopia	3
Ghana	4
Liberia	6
Rwanda	4
**Gender**	
Male	10
Female	7
**Position**	
Federal government	10
Regional government	4
Academic	3

**Figure 2:**
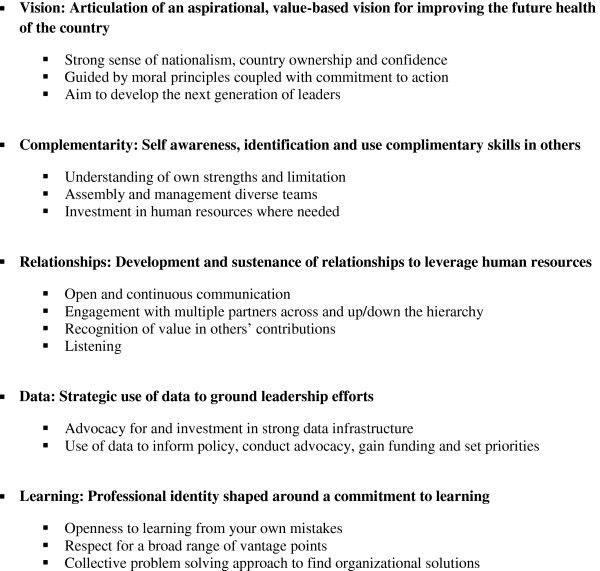
Five emergent themes.

Five key themes emerged as common to participants’ experiences in their leadership roles: having an aspirational, value-based vision for improving the future health of the country, being self-aware and having the ability to identify and use complementary skills of others, investing in and managing relationships, using data in decision making, and sustaining a commitment to learning (Figure 2). We describe these key themes with exemplary quotations from participants to illustrate each theme.

### Having an aspirational, value-based vision for improving the future health of the country

Participants articulated an aspirational, value-based vision for the future of their countries that was at once both optimistic and tempered by realism. This vision reflected a strong sense of nationalism, an imperative to develop the next generation of leaders, and guiding moral principles coupled with commitment to action. Historical context, including the consequences of living in a post-conflict environment, conditions of profound poverty, and current political struggles, framed their steadfast commitment to improving the health and well-being of their citizens. One participant noted the cultivation of future leaders as central to sustained stewardship of his country. In addition, the visions expressed by participants demonstrated strong sense of country ownership and confidence that they could improve health systems in their countries.

My generation decided to do something…for its own people…We had a lot of failures, but we were determined…So we have ideas that…are starting to stick, not in one generation, but they even prevail even after that generation is over. And we need time to cultivate them, to…convince everybody that our ideas are good ideas, that they are not personal, that they are good for the country…With confidence I’m telling you, we know the way out…We are here. (Ethiopian Participant #3).

For some, the vision for their country’s future was guided by moral underpinnings, as evidenced by the following participant’s declaration of his goal to ensure a core set of basic human rights:

It’s a systematic approach to uproot all those things that society doesn’t want… People should not be hungry. People should have access to basic education. People should have access to basic hygiene and basic medical supplies, services. (Liberian Participant #2).

And yet the espousal of principles alone was not sufficient; participants reflected that principles must be translated into action. This participant declared while beliefs such as fairness were essential to defining a vision, these beliefs must be coupled with practical action of benefit to every individual.

…you have to have general principles like fairness…But fairness can be a religion also. You can be dogmatic about it…So, beliefs are one thing. Actions are another thing…It is necessary to go from those fair, good beliefs, good ideas into practical things that test out, that go to every individual, every human being…(Ethiopian Participant #3).

### Being self-aware and having the ability to identify and use complementary skills in others

Participants reflected on their own strengths and limitations and the importance of seeking and using the complementary skills of others. One participant explicitly acknowledged that she “can’t know it all”, prompting her to seek the input of trusted others, particularly those “on the ground” who are closest to the issue.

…you can’t know it all…I’d rather involve everybody in the decision making because there’s something I might not know that somebody else knows…and then people feel a part of the whole process. Half of the time, I find solutions in people…they might know things that I wouldn’t know because of where I am….because they’re on the ground. (Rwandan Participant #1).

Self-awareness was coupled with assembling and managing diverse teams with complementary skills and investing human resources where needed. In the following example, the participant had a clear sense of his team’s goal, but also recognized the need for external support to accomplish this goal. He brought this expertise to the team from external sources, and despite initial resistance, peers gradually came to appreciate the value of diversity in teams.

…we’ve been able to achieve what we have achieved currently not only because of us…There are a whole lot of players involved…We did not have the capacity in country…we felt that maybe we could bring in people who could help…And in those initial days, the health workers in the Ministry of Health itself were not very welcoming because they felt we were just bringing in foreigners…they did not realize that these people were coming to work with them to be able to move a system forward. I think it is only now that people are actually appreciating that. (Liberian Participant #2).

### Tending to relationships

Developing and sustaining relationships with others was central to participants’ strategies for working in complex and resource-limited environments. These strategies included open and continuous communication with an emphasis on listening, and deliberate engagement and coordination of multiple partners, sometimes with competing interests. Participants described valuing the contributions of others as a means both to gather important input and to secure their support for a given initiative. Listening was regarded as necessary for effective problem solving as well as for the development of a trusting, communal, and collaborative environment.

You have to listen and don’t pretend to know it all. Sometimes we get the best ideas from the most unlikely sources of how to handle problems. You must give every…member of your group the chance to express themselves and through that process you learn and grow in your leadership roles and capacities…that is…the paradigm that I always carry along with me. (Rwandan Participant #3).

Participants perceived that investing in relationships by empowering others to voice their opinions and views provided additional benefits in terms of cultivating loyalty and support among others who could serve as allies in future efforts.

You let people speak…if you give people space to speak, you might be surprised how much they can actually contribute to the whole picture of leading something…when you do consider their opinions and they see you using their advice, you find them more trusting. (Rwandan Participant #1).

An emphasis on cultivating such engagement across many sectors of the community was common in participant descriptions of specific initiatives, such as national scale up of human immunodeficiency virus testing. One participant explained his view on the value of deliberate engagement of donor partners in the planning and implementation of programs, such as family planning, including building relationships at all levels, from the national to the local.

If you are working together, it is not only that partners will sit down and just say this is where we are. Also go down to the people and see where they are, how your support is helping…wherever we went, the district directors were really very pleased. Now, we are here saying, we now see why you are seeing quality partners, you’ll come and help us…And this relationship building, that starts from national, but it should not start there, it should go down. (Ghanaian Participant #1).

### Use of data in decision making

Participants discussed the critical role of data in their leadership efforts. Data was valued as essential to informing policy decisions, conducting advocacy, gaining external funding, and advancing one’s agenda within the country. Advocating for and investing in building a strong data infrastructure was a common experience. One participant recounted her frustrations with poor quality data related to mental health planning, and resolved to reform the national information system to produce valid metrics as a critical foundation for policy and advocacy efforts within the country.

The way of collecting statistics and data was…not standardized…we didn’t know how to use [the data] because we had poorly defined…the indicators to begin with. And the way we collected them from the district level up to the national level led to a lot of repetitions…And so we decided that if we were going to be able to do any advocacy for mental health, that we would have to reform the information system. (Ghanaian Participant #2).

Data were also used strategically with donor organizations and other partners. For example, this participant recounted his approach to using high quality data to carry out priority setting and long-term planning with partners.

…we'll present an organized and pre-prepared document…and prioritize the problems that were identified… in terms of social cost, economical cost, political commitments and the cost….they really want data and the figures. (Ethiopian Participant #2).

### Commitment to learning

Participants expressed commitment to learning as a core attribute of their professional identities in their leadership roles. This philosophy also included openness to learning from mistakes, and learning from both the front line and peers. Rather than blaming or scapegoating individual team members, participants used a collective approach to finding organizational solutions. One participant described this practice.

We sit together and we say, “Where did we go wrong?”…We don’t personalize it. We look after the thoughts, the ideas that led us astray. So, once we identify them, nobody is spared, the leaders, all of us from the top to the bottom. Once we are convinced that we have found the track that led us to failure, then we try to gather up momentum, to gather energy to find a solution, a way out. And that’s been the secret behind our success. (Ethiopian Participant #3).

Another shared his perspective on developing his leadership skills through daily interactions, with a view that learning is a continuous process.

You learn how to make your group dynamic leadership skills better each day dealing with a difficult team so the learning never stops. It never stops. Leadership is like science. So many new discoveries every day. (Rwandan Participant #3).

Respect for the value of a broad range of vantage points was commonly expressed, as reflected in this quotation from a participant who described learning from others with highly diverse backgrounds and experience.

A chief of a clinic must be part of the team, to put in his or her contribution. So in my work in life, I have learned from top people, doctors, nurses, nurse managers, and from the labourer. I have learned from [all of] them (Ghanaian Participant #1).

## Discussion

Participants in this study described several common aspects of their leadership experiences, ranging from the visionary to the pragmatic. Certain aspects are consistent with the empirical literature on leadership in high-income settings, such as having a clear vision [21-23]; commitment to learning across an organization [24,25]; attention to human relations in management [26,27]; and using data to guide decision making [21,28-30].

Nevertheless, other aspects of the health-care leadership experiences of our participants extend the findings of the limited existing empirical research in this area. Although previous literature suggests that, under conditions of stress, attention to relationships is often outweighed by task orientation [7,31,32], in our study, participants viewed investing in relationships as a core leadership function, even while working in chronically resource-constrained conditions. Participants did not focus extensively or exclusively on task-orientation or crisis management. Their commitment to relational aspects of their work was manifest in their openness to the views and experience of others, and persistent efforts to identify and fully engage individuals with complementary skills. Furthermore, participants described deliberate strategies for minimizing and addressing conflict, and for pursuing problem solving in productive and creative ways. They also expressed a balance between taking responsibility for achieving better health for the future of their country and accepting the interdependencies inherent in their roles; they did not perceive themselves as operating independently or in isolation from their broader environments and instead spent time developing relationships with sentinels inside and outside of their organizations whose feedback they could trust. They reflected on the complexity of their respective country contexts, and the need to be mindful of and responsive to multiple diverse constituencies and individuals within and external to the country. Participants also noted challenges associated with unpredictable or difficult political, economic and social conditions. In navigating these environments, they maintained broad, substantive, and prolonged engagement with others, particularly those they perceived as being on the front line, such as nurses district health officers in the community or staff in a health facility.

We did not find strong evidence of the use of data for accountability purposes, which was somewhat surprising, given the endorsement of using data for decision making and goal setting. Nonetheless, absent from the interviews were experiences and reflections on the use of data to monitor and correct or improve performance at any level. This absence may highlight both the practical challenges of poor and untimely data and cultural norms of limiting individual and group accountability, particularly in a public sector that lacks competition from effective market forces. The need to integrate data into corrective action and supportive supervision, while still maintaining the critical benefits of collaborative relationships, is a challenge for leadership capacity building within human resources for health.

What are the implications of our findings for current efforts to improve human resources for health in low income settings? Current models of capacity building address the need for core competencies such as technical capacities and management skills [6,33,34]. Our findings suggest that, while these skills are useful in leadership roles, competencies relevant to managing relationships, particularly in the context of increasing accountability, are also critical in the sub-Saharan African context. Efforts aimed at developing these skills in future leadership might include opportunities for experiential learning, close supervision and mentorship, and explicit focus throughout the system on the value of investing in and managing relationships. These skills are difficult to teach, particularly through externally developed programs, and will require more time and a deeper level of engagement than is typically invested in traditional efforts to strengthen health systems. Nevertheless, programmes that can be designed in true collaboration will be most successful; such programmes will recognize several paradoxes faced by people in health leadership roles in sub-Saharan Africa where needs are great and resources are limited. Based on our findings, leadership in these settings will require a careful balancing of several juxtapositions including country ownership and external input, aspirational vision for the future and practical action in the present, and strong collaborative relationships yet individual accountability. Rigorous research is needed to determine how such leadership capacity can be effectively developed, including strategies to facilitate within-country expertise and resources.

There are a number of strengths to this study. First, our purposeful sample included participants with diversity in country context, gender and leadership role. Second, we utilized a number of recommended strategies to insure rigor: consistent use of an interview guide; audio-taping and independent transcription; standardized coding and analysis; use of researchers with diverse racial, ethnic and professional backgrounds; and an audit trail to document analytic decisions [16,20,35,36]. Third, our high participation rate suggests that this is an issue that health-care leaders in sub-Saharan Africa are motivated to discuss, despite the personal and potentially sensitive nature of the topic.

Despite these strengths, our findings should also be interpreted in light of several limitations. First, because we used qualitative methods to understand the complex experiences of leaders in sub-Saharan African health care through purposeful sampling, our findings are exploratory and cannot be generalized to other leaders in sub-Saharan Africa. Second, our findings may have been influenced by social desirability bias, in which participants provide socially desirable responses [37]; however, we encouraged them to share personal experiences in detail and with candour, emphasizing our interest in both positive and negative aspects of their experience. Third, our study was geographically circumscribed to four countries in sub-Saharan Africa. These reflected both East and West Africa; nevertheless, other geographic regions may represent a substantially different environment for health-care leaders.

Consideration of historical, political, and sociocultural context is central to understanding African leadership [10-14]; however, little empirical work has been done to characterize leadership in this setting, particularly in health care. In this study, participant experiences of leadership in sub-Saharan Africa were characterized by persistent optimism and the ability to envision a better future, prioritization of investment in relationships despite conditions of high stress, and conscious use of data of all kinds to inform decisions. These insights can be useful in designing and implementing models to develop health-care leadership capacity in the region.

## Competing interests

The authors declare they have no competing interests.

## Authors’ contributions

Conception and design: LA Curry and EH Bradley; Analysis and interpretation of the data: LA Curry, L Taylor, P Chen, EH Bradley; Drafting of the article: LA Curry and L Taylor; Critical revision of the article for important intellectual content: LA Curry, L Taylor, P Chen, EH Bradley. All authors read and approved the final manuscript.
